# 4D Flow MRI Characterization of Left Atrial Vortical Flow in Atrial Fibrillation Following Catheter Ablation

**DOI:** 10.3390/jimaging12090441

**Published:** 2026-09-14

**Authors:** Omar Hassan, Hadi Hassan, Shuvam Prasai, Fiza Rajput, Julio Garcia

**Affiliations:** 1 Undergraduate Medical Education, Cumming School of Medicine, University of Calgary, Calgary, AB T2N 1N4, Canada; 2 Faculty of Medicine and Dentistry, University of Alberta, Edmonton, AB T6G 2E1, Canada; 3 Department of Radiology, Cumming School of Medicine, University of Calgary, Calgary, AB T2N 1N4, Canada; julio.garciaflores@ucalgary.ca; 4 Department of Cardiac Sciences, Cumming School of Medicine, University of Calgary, Calgary, AB T2N 1N4, Canada; 5 Stephenson Cardiac Imaging Centre, Libin Cardiovascular Institute, University of Calgary, Calgary, AB T2N 1N4, Canada; 6 Alberta Children’s Hospital Research Institute, Cumming School of Medicine, University of Calgary, Calgary, AB T2N 1N4, Canada

**Keywords:** atrial fibrillation, left atrium, vorticity, vortex flow, cardiac MRI, 4D flow MRI, hemodynamics, catheter ablation, imaging biomarkers

## Abstract

Atrial fibrillation (AF) is associated with abnormal left atrial (LA) blood flow patterns that contribute to thromboembolic risk. While blood flow stasis in AF has been extensively studied, the effects of AF and pulmonary vein isolation (PVI) on LA vortical flow remain incompletely understood. This study used four-dimensional flow magnetic resonance imaging to quantify LA vorticity and vortex volume in 107 patients with AF before PVI, 56 patients after PVI, and 19 non-AF controls. Vortex structures were identified using the Lambda2 method, and group differences were assessed after adjustment for age and sex. Pre-ablation patients demonstrated significantly lower LA vorticity than both post-ablation patients and controls, whereas post-ablation patients did not differ from controls. In contrast, mean and maximum LA vortex volumes remained elevated in both AF groups relative to controls, with no significant differences between pre- and post-ablation patients. These findings indicate that PVI is associated with improvements in LA vorticity but not vortex volume. The persistence of enlarged LA vortices despite normalization of vorticity suggests that these metrics capture distinct aspects of atrial remodeling and may provide complementary insight into AF-related pathophysiology.

## 1. Introduction

Atrial fibrillation (AF) is a burdensome cardiac arrhythmia, impacting more than 52 million people globally [1]. Non-invasive imaging approaches, such as four-dimensional (4D) flow magnetic resonance imaging (MRI), have revealed that AF is characterized by aberrant blood flow patterns within the heart, including increased blood flow stasis and slower blood velocities within the left atrium (LA) [2,3]. This results in prothrombotic conditions that are known to contribute to embolic stroke in AF [4].

While stagnant blood flow is perhaps the most well-studied hemodynamic consequence arising from AF pathophysiology, and an established contributor to subsequent thromboembolic complications, there exist other hemodynamic markers that can be non-invasively visualized and may offer unique insight into intracardiac flow [5]. One such construct is vorticity, defined as the extent to which a fluid exhibits local rotational motion [6]. The arrangement of flow into vortical patterns is thought to support efficient transfer of blood between cardiac chambers while minimizing energy loss [7]. This idea is primarily supported by the literature examining left ventricular flow, wherein vortices originate at the mitral valve during diastole and are efficiently redirected to the aorta during systole [7,8]. However, research has also examined vortex formation and functionality within the LA [9,10]. Here, vortices are thought to form as a result of blood inflow to the LA from opposing pulmonary veins, and they function to minimize energy loss and prevent LA stasis [9,10,11]. Given that AF as a condition is characterized by perturbed blood flow dynamics within the LA [2,3], it becomes logical to consider the phenomenon of vortices within this context.

Prior studies have indeed sought to examine vortical flow in the context of AF. Garcia et al. (2020) demonstrated that individuals with AF present with larger LA vortices, and that greater LA vortex volumes are associated with higher CHA_2_DS_2_-VASc scores (indicating greater stroke risk) [12]. Corroborating this finding, Spartera et al. (2022) revealed that decreased LA vorticity predicts higher incidence of embolic stroke in individuals with AF [13]. Crucially, this significant association persists even when controlling for CHA_2_DS_2_-VASc scores [13], suggesting that integration of vorticity may offer greater utility for stroke risk stratification than the widely used CHA_2_DS_2_-VASc score alone.

Further investigations have sought to determine the impact of clinical intervention on vortical flow in AF. For instance, Spartera et al. (2021) demonstrated that individuals experiencing active AF display larger LA vortex volumes and lower vorticity than individuals in sinus rhythm, and crucially, this difference is abolished after cardioversion-induced restoration of sinus rhythm in AF participants [14]. While this finding suggests that rhythm restoration may correct LA hemodynamics, cardioversion is often only effective in the short-term, with nearly half of all patients undergoing this procedure experiencing arrhythmia recurrence within just three months [15]. This therefore substantiates examination of how more durable therapeutic interventions to correct AF (e.g., catheter ablation) may impact vortical flow, given that pulmonary vein isolation (PVI) ablation has been shown to improve other hemodynamic markers (e.g., reduced LA stasis) in previous work [3,16].

In one such investigation, Vogl et al. (2022) reported no consistent effect of catheter ablation on LA vorticity in patients with AF [17]. However, this study had a limited sample size (ten participants), and relied on computational modelling that is predicated upon several assumptions which may compromise validity of the results [18]. This warrants further investigation with larger sample sizes and in vivo imaging of cardiac flow. To this end, the present study aimed to utilize 4D flow MRI to investigate LA vortical flow in individuals with AF, and to characterize the effect of PVI on this hemodynamic phenomenon.

## 2. Materials and Methods

### 2.1. Study Population

A total of 214 participants were retrospectively selected for this study using the Cardiovascular Imaging Registry of Calgary (CIROC). The study population consisted of a non-AF control group, a pre-ablation group comprising patients with paroxysmal AF who had been diagnosed more than two years prior to enrollment, and a post-ablation group who had undergone PVI between 3 and 6 months before their imaging examination. Participant consent, health questionnaires, and MRI-related variables were collected using a commercial electronic data capture platform (Acuity^®^, Cohesic Inc., Calgary, AB, Canada).

Patients in both the pre-ablation and post-ablation cohorts were required to be at least 18 years of age, in sinus rhythm at the time of imaging, and have no greater than mild mitral valve regurgitation. Additional exclusion criteria included cardiomyopathy, complex congenital heart disease, implanted cardiac devices, severe renal dysfunction (estimated glomerular filtration rate ≤ 30 mL/min/1.73 m^2^), or any recognized contraindication to MRI. Control participants were required to be at least 18 years old and have no known history of cardiovascular disease, diabetes mellitus, or uncontrolled hypertension.

**Figure 1:**
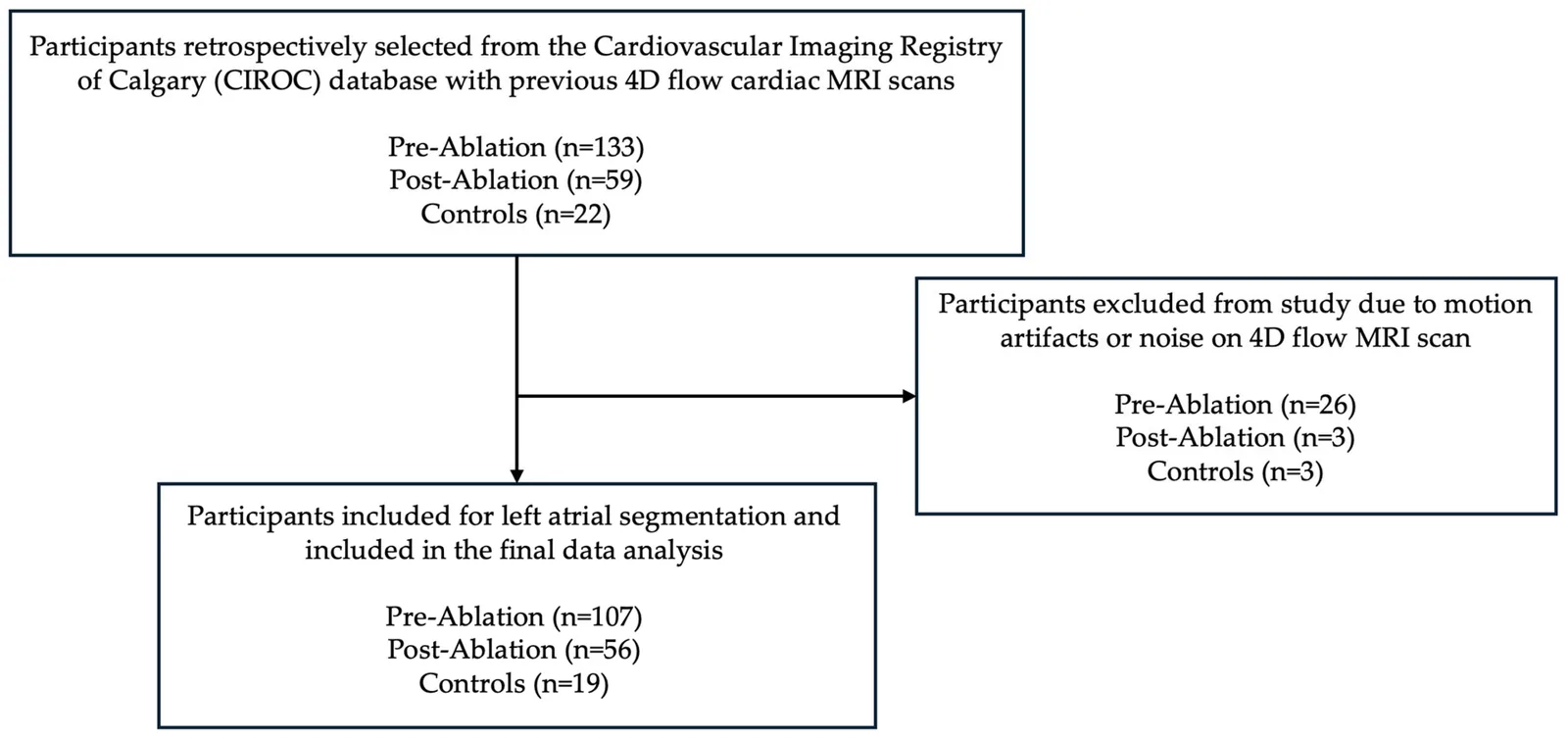
Flow diagram depicting selection of participants and exclusion due to insufficient imaging quality, giving rise to the final study sample.

All imaging datasets underwent quality assessment by an experienced cardiovascular MRI reader. Following review, datasets with substantial motion artifact or image degradation that prevented reliable analysis were excluded, such that the final analysis included 107 pre-ablation, 56 post-ablation, and 19 control participants (Figure 1).

### 2.2. Cardiac Magnetic Resonance Imaging Protocol

All MRI examinations were performed using 3.0-T clinical scanners (Skyra and Prisma; Siemens Healthineers, Erlangen, Germany). A standardized imaging protocol was applied to all participants, as previously described [3,12,19]. Cine images were acquired in standard long-axis orientations, including two-, three-, and four-chamber views, in addition to a contiguous short-axis stack encompassing the entire left ventricle.

Following intravenous administration of a gadolinium-based contrast agent (0.2 mmol/kg Gadovist^®^, Bayer Inc., Mississauga, ON, Canada), a three-dimensional contrast-enhanced magnetic resonance angiogram was obtained to visualize LA and pulmonary venous anatomy. Subsequently, whole-heart 4D flow MRI (Siemens WIP 785A) was acquired over approximately 5 to 10 min to characterize intracardiac blood flow dynamics throughout the cardiac cycle [3,12,19].

### 2.3. Conventional Cardiac Image Analysis

Conventional cardiac MRI images were analyzed for assessment of cardiac chamber volumes, which was performed using commercially available software (cvi42; Circle Cardiovascular Imaging Inc., Calgary, AB, Canada).

### 2.4. 4D Flow MRI Analysis

All 4D flow MRI datasets underwent standardized preprocessing, including correction for Maxwell phase effects, eddy current-related phase offsets, and velocity aliasing when present. A phase-contrast magnetic resonance angiography dataset was generated from each acquisition and used to define LA anatomy. LA segmentation was performed using a custom software package developed in MATLAB (R2024b; MathWorks, Natick, MA, USA), according to the anatomical boundaries that could be reliably delineated from the acquired images. For this analysis, the LAA was considered part of the LA region and was included to the extent that it could be reliably delineated. The resulting LA segmentation was applied as a three-dimensional mask to isolate blood flow within the atrium.

Interobserver reproducibility was evaluated by having two trained analysts independently perform segmentation and quantitative flow analysis under the supervision of an experienced cardiac MRI reader. Image datasets were initially analyzed independently and then exchanged for repeat processing by the second analyst. Agreement between observers was assessed using blood flow velocities, with differences below 5% considered acceptable.

LA vortex structures were identified using the Lambda2 (λ_2_) vortex detection method, a well-established fluid dynamics technique for objectively characterizing three-dimensional vortical flow [20]. Briefly, spatial velocity field gradients were calculated from the 4D flow velocity field using three-dimensional compact Richardson interpolation, which helps reduce local noise and partial volume effects. The λ_2_ parameter was subsequently derived, with negative λ_2_ values representing coherent vortex regions. Because not all negative λ_2_ regions necessarily represent coherent vortex structures, a patient-specific λ_2_ threshold was subsequently applied.

**Figure 2:**
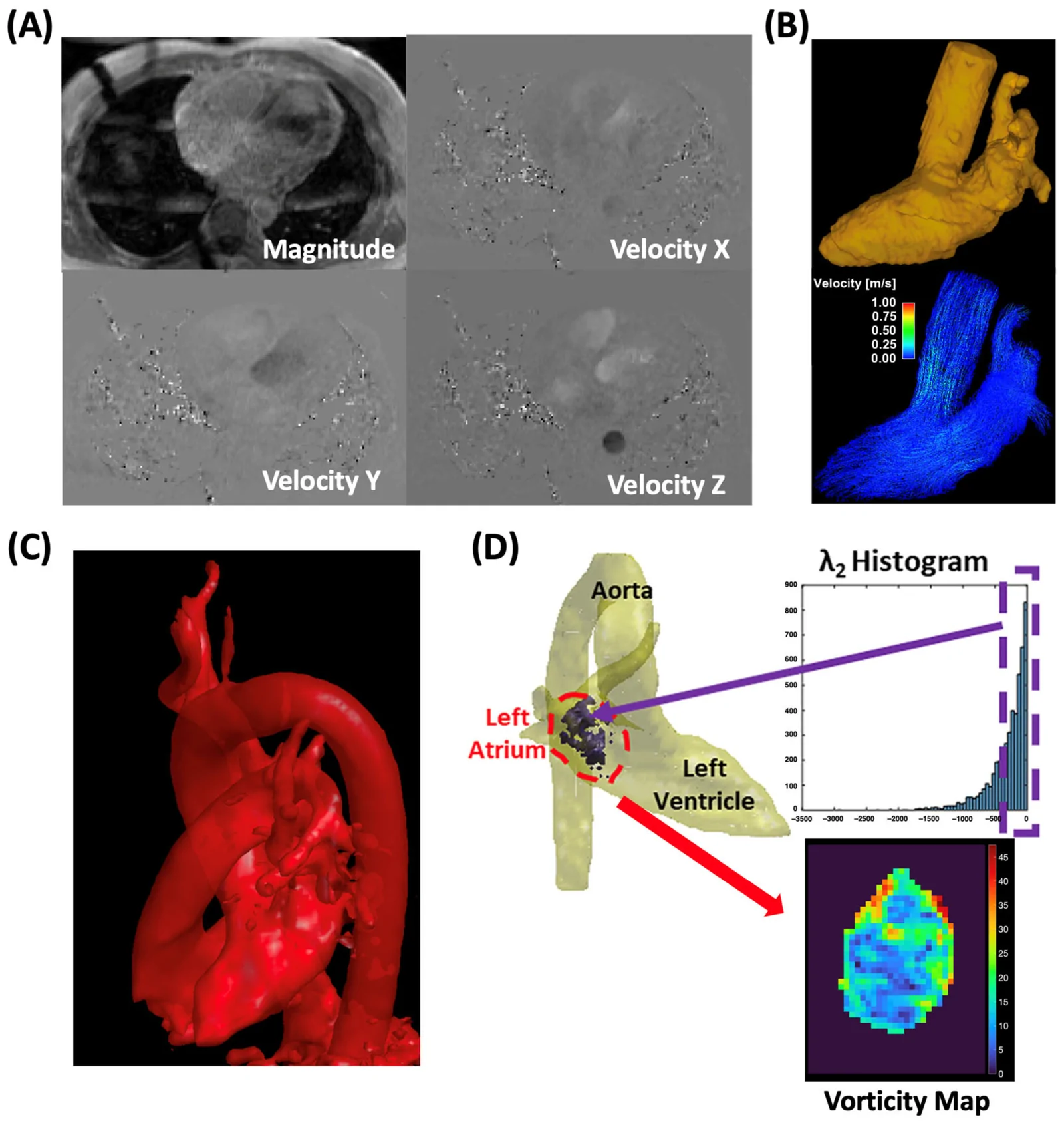
Summary of steps involved in data preparation and image analysis: (**A**) Sample images derived from 4D flow MRI following correction protocol. (**B**) Visualization of left heart blood flow dynamics. (**C**) Time-averaged rendering of phase-contrast magnetic resonance angiography, from which the left atrium (LA) is manually demarcated in successive sections to obtain a 3D segmentation. (**D**) Vortex volumes derived from LA segmentations using the λ_2_ method, and vorticity values obtained by averaging LA vorticity over the cardiac cycle. Panel D adapted with permission from Ref. [12]. Copyright © 2019, International Society for Magnetic Resonance in Medicine, published by Wiley Periodicals, Inc.

To account for inter-patient variation in flow characteristics, a patient-specific λ_2_ threshold was determined by generating a histogram of λ_2_ values obtained throughout the cardiac cycle and defining the threshold as one-half of the mean λ_2_ value [12]. Voxels satisfying this threshold criterion were then retained for subsequent connectivity-based region analysis and vortex quantification. Only vortex regions exceeding 75 mm^3^ were retained, and neighboring voxels were required to share at least four connected voxels to be classified as belonging to the same vortex structure. Vortex volume was quantified as the total volume of identified vortex regions and reported in cubic centimeters (cm^3^). Maximum vortex volume was defined as the largest vortex volume at any single point during the cardiac cycle, and mean vortex volume was computed by time-averaging vortex volumes across the entire cardiac cycle. Similarly, mean vorticity was computed by time-averaging whole-LA vorticity values across the cardiac cycle, and reported in inverse seconds (s^−1^). An overview of the 4D flow MRI analysis is summarized in Figure 2.

Demographic and clinical variables used in subsequent analyses were obtained from study questionnaires, imaging records, and linked clinical databases in accordance with institutional research ethics approval.

### 2.5. Statistical Analysis

Continuous variables are reported as mean ± standard deviation, and categorical variables are presented as frequencies and percentages. A main-effects univariate general linear model (i.e., analysis of covariance [ANCOVA]) was used to compare vortical flow outcomes (time-averaged vorticity, maximum vortex volume, mean vortex volume) across treatment groups (pre-ablation, post-ablation, or control), adjusting for age and sex. Treatment group and sex were entered as fixed factors, and age was entered as a continuous covariate.

Adjusted estimated marginal means were calculated for each study group after controlling for age and sex, and post-hoc pairwise comparisons were Bonferroni-adjusted. Statistical significance was defined as a two-sided *p*-value < 0.05. All statistical analyses were performed using IBM SPSS Statistics version 29 (IBM Corp., Armonk, NY, USA).

## 3. Results

### 3.1. Patient Demographics

Demographic characteristics for individuals in the pre-ablation, post-ablation, and non-AF control group are reported in Table 1. The three groups were generally similar on each of the reported characteristics with the exception of age, where non-AF controls were considerably younger (37 ± 13 years) than pre-ablation (60 ± 11 years) and post-ablation (61 ± 9 years) participants.

### 3.2. Left Atrial Vorticity

**Figure 3:**
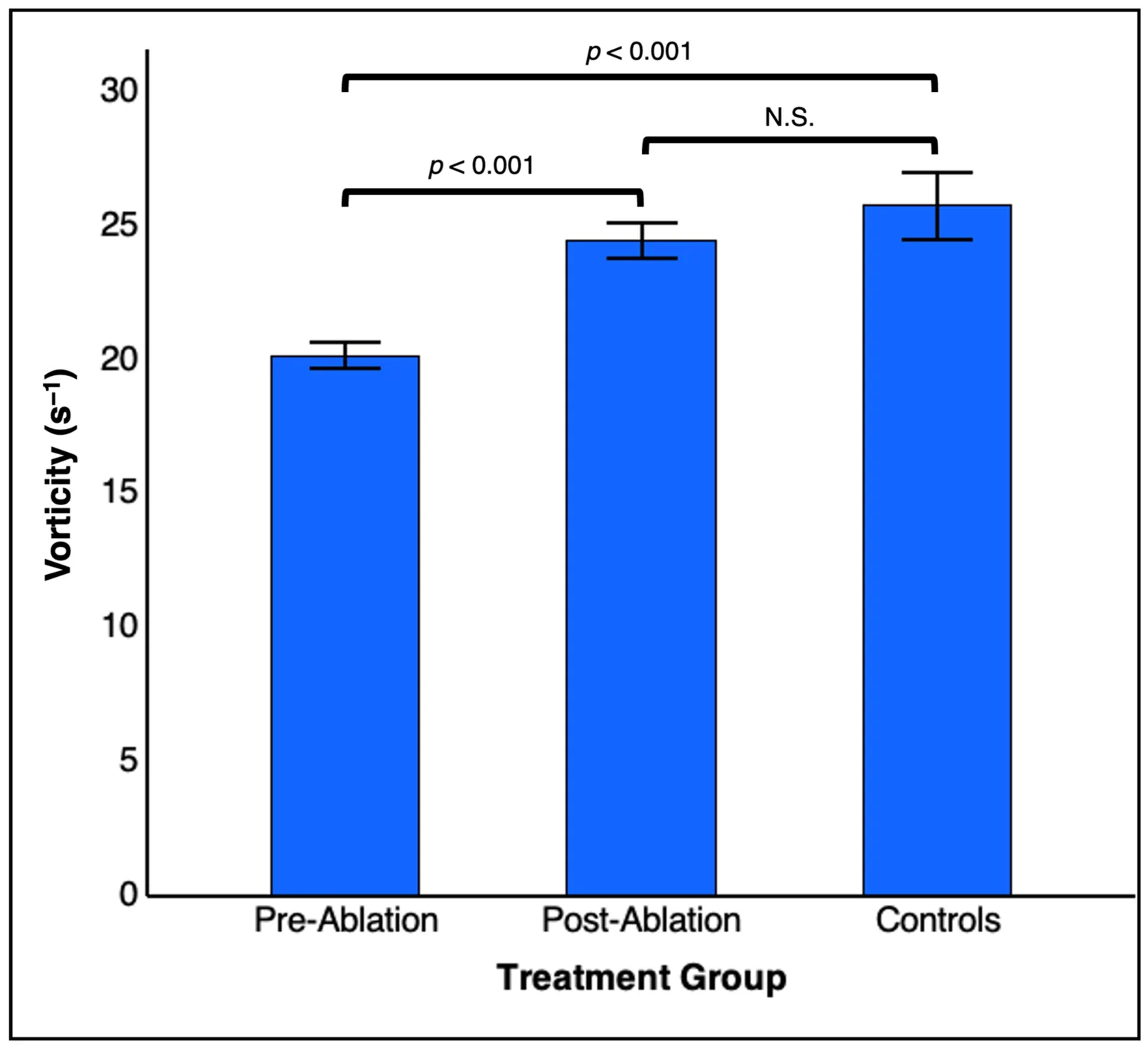
Time-averaged LA vorticity values in pre-ablation, post-ablation, and control participants. Data shown are estimated marginal means adjusted for age and sex. Error bars represent ± 1 standard error of the mean (SEM).

Pre-ablation patients demonstrated lower LA vorticity values (19.92 ± 4.64 s^−1^) than post-ablation participants (24.18 ± 4.63 s^−1^), who had similar LA vorticity to non-AF controls (28.56 ± 5.91 s^−1^). After adjustment for age and sex, there was a significant main effect of treatment group on LA vorticity, F(2, 177) = 20.559, *p* < 0.001, partial η^2^ = 0.19 (Figure 3). Post-hoc pairwise comparisons of estimated marginal means revealed that pre-ablation patients had significantly lower adjusted LA vorticity values than post-ablation patients (mean difference = −4.29, 95% CI [−6.16, −2.42], *p* < 0.001) and non-AF controls (mean difference = −5.58, 95% CI [−8.96, −2.20], *p* < 0.001). No significant differences in LA vorticity were observed between post-ablation patients and non-AF controls.

**Figure 4:**
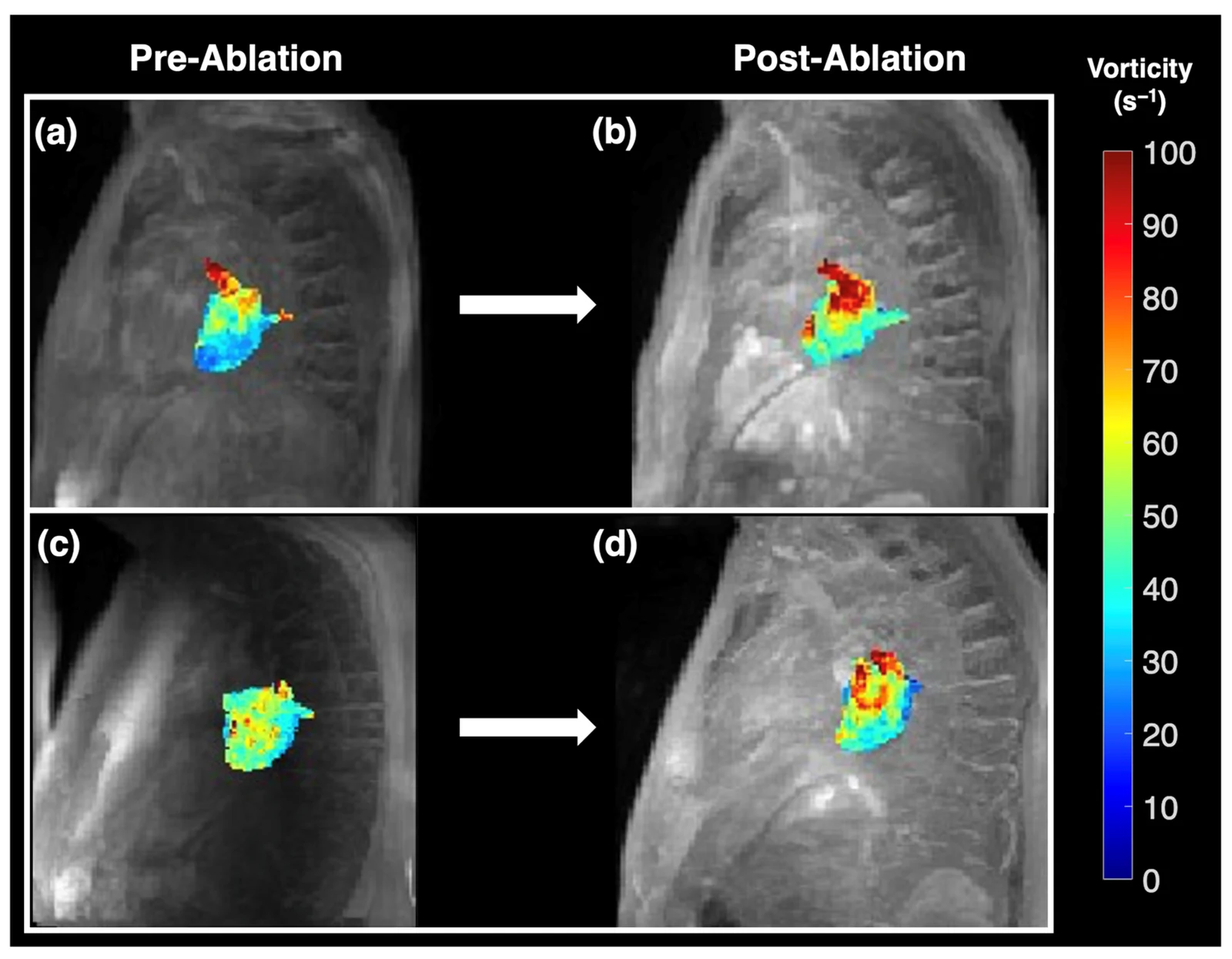
Representative visualizations of vorticity before and after pulmonary vein isolation in a 62-year-old male (Panels (**a**,**b**)) and 59-year-old male patient (Panels (**c**,**d**)) with paroxysmal atrial fibrillation. Images represent maximum intensity projections of vorticity in 3D left atrial segmentations, with warmer colours corresponding to greater vorticity values.

Representative visualizations of LA vorticity prior to and following catheter ablation in selected patients are provided in Figure 4.

### 3.3. Left Atrial Vortex Volumes

**Figure 5:**
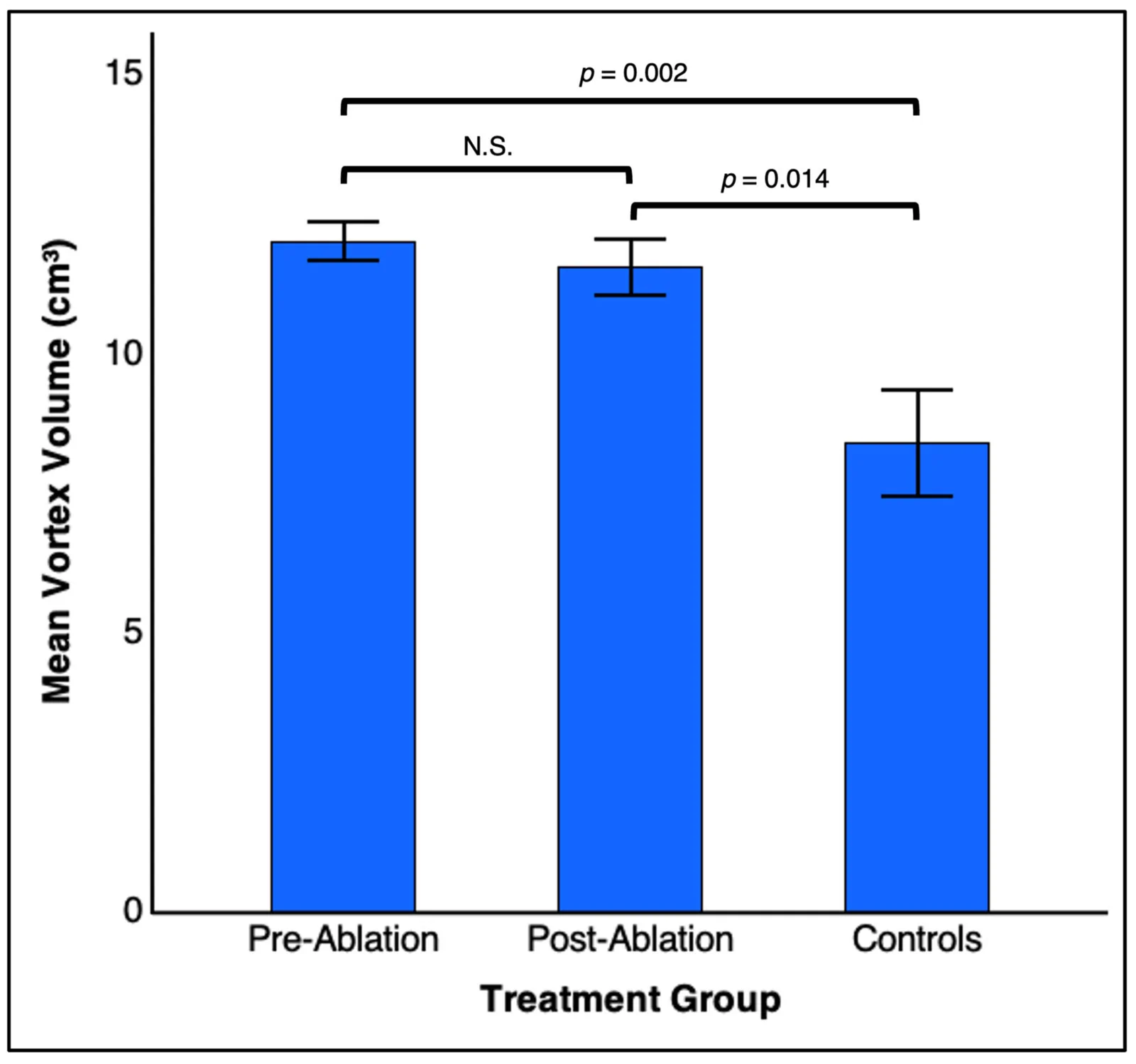
LA vortex volumes averaged across the cardiac cycle in pre-ablation, post-ablation, and control participants. Data shown are estimated marginal means adjusted for age and sex. Error bars represent ± 1 SEM.

Additionally, pre-ablation participants had similar mean LA vortex volumes (12.43 ± 3.42 cm^3^) to post-ablation participants (12.33 ± 3.78 cm^3^), both of which were larger than those found in non-AF controls (7.08 ± 2.42 cm^3^). After adjustment for age and sex, there was a significant main effect of treatment group on mean LA vortex volume, F(2, 177) = 6.17, *p* = 0.003, partial η^2^ = 0.07 (Figure 5). Post-hoc pairwise comparisons of estimated marginal means revealed no significant differences in mean LA vortex volume between pre-ablation and post-ablation patients (mean difference = 0.46, 95% CI [−0.89, 1.80], *p* = 1.00). However, relative to non-AF controls, adjusted mean LA vortex volumes were significantly higher in both pre-ablation patients (mean difference = 3.62, 95% CI [1.12, 6.13], *p* = 0.002) and post-ablation patients (mean difference = 3.17, 95% CI [0.50, 5.83], *p* = 0.014).

**Figure 6:**
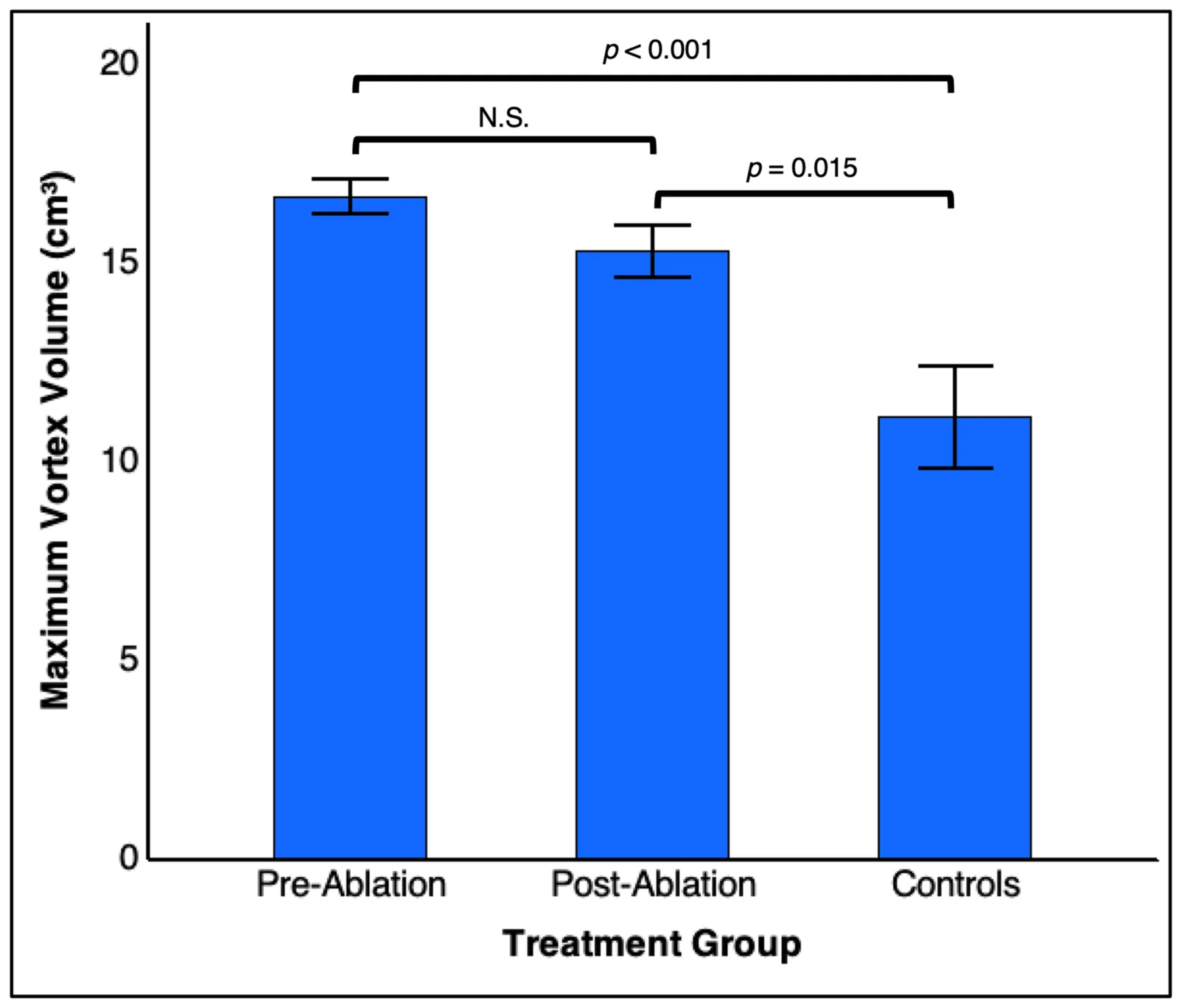
Maximum LA vortex volumes across the cardiac cycle, in pre-ablation, post-ablation, and control participants. Data shown are estimated marginal means adjusted for age and sex. Error bars represent ± 1 SEM.

Finally, maximum LA vortex volumes were similar in pre-ablation (17.15 ± 4.66 cm^3^) and post-ablation patients (16.23 ± 4.83 cm^3^), which were both larger than those in non-AF controls (10.11 ± 3.01 cm^3^). After adjustment for age and sex, there was a significant main effect of treatment group on maximum LA vortex volume, F(2, 177) = 8.81, *p* < 0.001, partial η^2^ = 0.09 (Figure 6). Post-hoc pairwise comparisons of estimated marginal means revealed that pre-ablation patients had significantly higher maximum vortex volumes than non-AF controls (mean difference = 5.55, 95% CI [2.20, 8.91], *p* < 0.001), as did post-ablation patients (mean difference = 4.20, 95% CI [0.62, 7.71], *p* = 0.015). Post-ablation maximum vortex volumes were unchanged relative to those in pre-ablation patients (mean difference = −1.36, 95% CI [−3.16, 0.44], *p* = 0.21).

## 4. Discussion

In the present study, we sought to characterize the impact of PVI on LA vortical flow in individuals with AF, using 4D flow MRI. To this end, we first demonstrated decreased LA vorticity in individuals with AF prior to PVI, relative to non-AF controls. This finding contrasts with prior work suggesting no difference in LA vorticity between AF participants and non-AF controls [21], a discrepancy that likely derives from the larger sample size analyzed in the present study. Importantly, this alteration of LA hemodynamics may contribute to the elevated stroke risk observed in this population. In particular, it is thought that strong vortical flow within the LA enables reconciliation of oppositional pulmonary venous inflows in a manner that minimizes the loss of fluid kinetic energy, hence preserving blood flow velocity and enabling efficient emptying of the LA to prevent blood flow stasis [9,10,11]. When vortex formation is disrupted, more energy is lost in the collision of pulmonary venous inflow, which slows LA blood velocities and promotes stasis [9,10,11], a causal contributor to stroke risk [4].

As a therapeutic intervention to correct AF, the present results demonstrate that LA vorticity levels in post-ablation patients are normalized to levels observed in non-AF controls. This finding aligns well with the literature that demonstrates improvements in LA structure and hemodynamics following ablation in AF [22], which are suggested to contribute to the decrease in stroke risk attributed to this procedure [23]. However, this finding conflicts with the prior work of Vogl et al. (2022), wherein no significant effect of catheter ablation was observed [17]. This can likely be explained by the larger sample size of the present study, as well as the more physiologically relevant determination of in vivo hemodynamics using 4D flow MRI [18] in the present work, as opposed to the use of computational fluid dynamics in Vogl et al. (2022) [17].

Although LA vorticity was normalized after ablation, both maximum and mean LA vortex volumes were unchanged after PVI, remaining elevated in both pre- and post-ablation patients, relative to controls. This failure of vortex volumes to normalize after ablation is interesting, especially when considering that overall LA chamber volumes were numerically reduced to control levels in post-ablation patients (Table 1). This suggests that increased LA vortex volumes in AF, also observed in Garcia et al. (2020), are not simply attributable to LA dilation [12]. Similarly, both pre- and post-ablation patients underwent 4D flow MRI in sinus rhythm, and therefore, the persistent enlargement of LA vortices relative to controls cannot be attributed to (recurrence of) active fibrillation of the atria during image acquisition. Instead, the maintenance of large LA vortices is likely a reflection of the multitude of LA structural and functional alterations that characterize AF [22,24], which often persist even after ablation [22].

This finding underscores the utility of leveraging the variety of hemodynamic markers available through 4D flow MRI, and not simply restricting one’s consideration to the canonical LA blood flow stasis [5]. For instance, although post-ablation patients demonstrate normalized LA blood flow stasis [3]—and presently, time-averaged vorticity—comparable to non-AF levels, clinical practice guidelines recommend that anticoagulation be continued after ablation of AF, particularly in individuals with high CHA_2_DS_2_-VASc scores [25]. This practice implies that despite the apparent normalization of LA stasis and vorticity after successful ablation [3], individuals with AF remain at higher stroke risk relative to the non-AF population [25]. Therefore, LA blood flow stasis and time-averaged vorticity are, at best, incomplete indices of thromboembolic risk in this population. In contrast to these markers, the fact that LA vortex volumes remain elevated in post-ablation participants in the present study suggests that, in principle, larger LA vortices may independently contribute to the presumed increased risk of thromboembolism in these patients. However, a causal relationship between LA vortex volume and subsequent embolic stroke risk has not been identified, and alternative explanations must be considered. For instance, Garcia et al. (2020) postulated that large vortices in a normal-sized LA (e.g., after PVI-induced remodeling) in fact offer a protective function, by washing out stagnant blood flow across the atrium [12]. Given these competing possibilities, future work should aim to more comprehensively characterize the mechanistic relationship of LA vortex volume to AF pathophysiology and complications.

## 5. Limitations

There are certain shortcomings of the present study which future work should strive to build upon. One such limitation is the failure to consider hemodynamics of the LAA separately from the LA body. The LAA is the primary thrombogenic locale in AF [26]. Prior echocardiographic work has demonstrated weaker LAA vortices in the context of AF [27], and computational simulations have shown that vortex characteristics within the LA body directly influence the degree of LAA blood flow stasis [28]. These findings highlight the importance of analyzing the LAA separately in the context of vortical flow, but the present study’s lack of stratification of these structures precludes such precise analysis.

Additionally, all participants were imaged in sinus rhythm at the time of MRI acquisition. Consequently, the present study cannot evaluate the acute effects of AF on LA vortical flow. Instead, the findings reflect differences in hemodynamic characteristics among individuals with a history of AF before and after PVI while in sinus rhythm. Future studies incorporating imaging during active AF may help further distinguish the transient hemodynamic effects of arrhythmia from the more persistent structural and functional alterations associated with the disease.

Furthermore, the present study considered one intervention, specifically PVI, for AF. However, there exist a number of modalities by which PVI ablation can be performed, with varying atrial structural and functional consequences with no clear superiority of outcome [22]. Furthermore, computational simulations of other procedures such as LAA occlusion have been shown to improve LA hemodynamics in AF [29], but rigorous testing of this mechanism in vivo using 4D flow MRI remains an open question [30]. Therefore, future investigations could consider analysis of changes in vortical flow, alongside other 4D flow derived metrics, for patients undergoing a larger variety of procedures aimed at treating AF.

An important limitation relates to the composition of the control cohort. Control participants were substantially younger than individuals in both AF groups and the age distributions demonstrated limited overlap. Although age was included as a covariate in all analyses, statistical adjustment cannot fully replace the use of age-matched controls, and residual confounding may remain. Furthermore, the control cohort was relatively small compared with the AF groups, which may limit the precision of healthy reference estimates. Future studies should include larger, age-matched control populations to confirm the present findings and better define normative values for LA vortical flow metrics.

Finally, with respect to participant recruitment, only a limited number of AF patients were followed (i.e., matched) in the pre- and post-ablation conditions, and therefore individual differences may confound the treatment effect. This is especially relevant to consider, as it has been established that LA hemodynamics (e.g., blood flow stasis) vary significantly in relation to individual-level demographic and clinical characteristics [31,32]. This also substantiates future inquiry into which individual factors are associated with alterations in LA vortical flow.

Furthermore, the single-center nature restricts generalizability of the study, and future work should recruit a more diverse population across multiple centers to enhance external validity [33].

## 6. Conclusions

The present study used 4D flow MRI to characterize LA vortical flow in individuals with AF, before and after PVI. Patients with AF demonstrated reduced LA vorticity relative to non-AF controls, while vorticity in post-ablation patients was comparable to that of controls. These findings suggest that catheter ablation is associated with improvements in this aspect of LA flow, and further support the concept that AF is characterized by measurable alterations in intracardiac hemodynamics.

In contrast, mean and maximum LA vortex volumes remained elevated following ablation and did not differ from those observed in pre-ablation patients. This persistence of enlarged vortices despite normalization of vorticity suggests that these metrics reflect distinct features of LA flow remodeling and may provide complementary information regarding the hemodynamic consequences of AF. Future studies should further investigate the mechanistic significance of vortex volume, particularly with respect to thromboembolic risk, and its potential value alongside established 4D flow MRI markers in hemodynamic assessment of individuals with AF.

## Figures and Tables

**Table 1:** Demographic characteristics of participants included in the final analysis, summarized by group. Continuous data are presented as mean ± standard deviation, and sample sizes are indicated for specific measures where number of observations differs from total group size, due to missing participant data.

Characteristic	Pre-Ablation (*n* = 107)	Post-Ablation (*n* = 56)	Controls (*n* = 19)
Age, years	60 ± 11	61 ± 9	37 ± 13
Sex, female	34.6%	16.1%	42.1%
Body Mass Index, kg/m^2^	29 ± 5	28 ± 5	25 ± 5
Heart Rate, bpm	63 ± 13	67 ± 12	65 ± 12
Left Ventricular Ejection Fraction, %	60 ± 7 (*n* = 105)	60 ± 5	64 ± 4
Left Atrial Volume Index, mL/m^2^	42 ± 14 (*n* = 101)	37 ± 11 (*n* = 54)	37 ± 8

## Data Availability

The anonymized data presented in this study are available upon reasonable request and subject to a data-sharing agreement. The data is not publicly available due to privacy and ethical restrictions.
